# Synchrotron Mössbauer spectroscopy using high-speed shutters

**DOI:** 10.1107/S090904951003863X

**Published:** 2010-11-23

**Authors:** T. S. Toellner, E. E. Alp, T. Graber, R. W. Henning, S. D. Shastri, G. Shenoy, W. Sturhahn

**Affiliations:** a Argonne National Laboratory, 9700 South Cass Avenue, Argonne, IL 60439, USA; b Center for Advanced Radiation Sources, The University of Chicago, Chicago, IL 60637, USA

**Keywords:** Mössbauer spectroscopy, nuclear resonant scattering, X-ray shutter

## Abstract

A new method of performing Mössbauer spectroscopy with synchrotron radiation is demonstrated that involves using a high-speed periodic shutter near the focal spot of a microfocused X-ray beam. This fast microshuttering technique operates without a high-resolution monochromator and has the potential to produce much higher signal rates. It also offers orders of magnitude more suppression of unwanted electronic charge scattering. Measurement results are shown that prove the principle of the method and improvements are discussed to deliver a very pure beam of Mössbauer photons (*E*/Δ*E* ≃ 10^12^) with previously unavailable spectral brightness. Such a source will allow both Mössbauer spectroscopy in the energy domain with the many advantageous characteristics of synchrotron radiation and new opportunities for measurements using X-rays with ultra-high energy resolution.

## Abstract 2

A new method of performing Mössbauer spectroscopy using a fast shutter in combination with microfocused synchrotron radiation is demonstrated.

## 1. Introduction

Synchrotron Mössbauer spectroscopy (SMS) provides information on atomic environments by measuring the interaction between nuclear moments and the local electric and magnetic fields. It does this by exciting low-energy (∼10–100 keV) nuclear resonances with synchrotron radiation and detecting the coherent emission as a function of time (time-domain measurements) or as a function of incident X-ray energy (energy-domain measurements). Time-domain measurements are much more common and have relatively short data collection times (typically <1 h), while the latter require the production of an ultra-high-energy-resolution X-ray beam (∼10 neV) (Chumakov *et al.*, 1990 ▶; Smirnov *et al.*, 1997 ▶; Mitsui *et al.*, 2007 ▶) and data collection can take many hours per spectrum. There is also a mixed class of measurements employing time discrimination in a manner that yields an energy spectrum without requiring an ultra-high-resolution X-ray beam. These alternative procedures may be direct (Seto *et al.*, 2009 ▶) or rely on algorithms to reconstruct an energy spectrum from a multitude of time spectra (Coussement *et al.*, 1996 ▶; L’abbé *et al.*, 2000 ▶; Sturhahn *et al.*, 2003 ▶; Callens *et al.*, 2003 ▶). Data collection times to acquire adequate statistics for these methods are also typically hours. It would be a significant advance if one could improve signal rates and perform both time-domain and energy-domain measurements in the same set-up with the source characteristics of synchrotron radiation: a polarized beam with a small cross-sectional size and high spectral-brightness. This would allow, for instance, nuclear resonant diffraction from select nuclear transitions to obtain useful site-selective structural information (Stephens & Fultz, 1997 ▶) with much greater practicality. Obtaining complementary energy spectra along with time spectra can also help to unambiguously determine hyperfine parameters for complex materials with multiple sites and complicated field distributions. Performing both types of measurements efficiently would require one to detect the nuclear resonant scattering with minimal losses while completely suppressing the electronic charge scattering.

Ever since the suggestion to use synchrotron radiation as a source to perform Mössbauer spectroscopy (Ruby, 1974 ▶), experimentalists have considered various means to tackle this primary technical problem of observing an extremely narrow energy resonance using a broadband X-ray source. Nuclear resonant scattering is distinct from non-resonant electronic charge scattering by way of its sensitivity to hyperfine interactions, which exhibits a strong polarization dependence, and its long excited-state lifetime (∼100 ns). Numerous methods to separate the nuclear resonant signal from the vast amount of electronic scattering have been employed including, for example, pure nuclear Bragg reflections (Gerdau *et al.*, 1985 ▶; Smirnov, 2000 ▶), anti-reflecting nuclear-resonant films (Röhlsberger *et al.*, 1992 ▶; Röhlsberger, 1999 ▶), crossed linear-polarizers (Siddons *et al.*, 1993 ▶; Toellner *et al.*, 1995 ▶) or the nuclear-lighthouse effect (Röhlsberger *et al.*, 2000 ▶). The most common method has been to take advantage of the long excited-state lifetime to separate the delayed nuclear resonant scattering from the prompt electronic charge scattering using a fast-timing detector and time-filtering methods. The best detectors for this purpose are avalanche photodiode detectors (APD) (Kishimoto, 1992 ▶; Baron *et al.*, 2006 ▶), but they are unable to operate under the enormous X-ray load of eV-bandwidth synchrotron radiation. One typically reduces the X-ray load by orders of magnitude by using a high-resolution monochromator (HRM) to reduce the bandwidth of the synchrotron radiation to around 1 meV (Toellner, 2000 ▶). For an efficient 1 meV monochromator at a third-generation synchrotron source, this results in an X-ray load (10^10^ photons s^−1^) that still overwhelms fast-timing detection systems. One usually relies on sample absorption, additional detectors, HRM inefficiency and additional absorbers to reduce the X-ray load further so that the detection system can operate properly. HRMs have spectral efficiencies that are typically 5–50%, but can be lower. Theoretically, reducing the bandwidth of an HRM could improve the signal-to-background ratio, but this becomes increasingly more difficult and usually with less efficiency in practice. A method that could suppress more electronic scattering and circumvent HRM inefficiency could potentially improve signal rates by one to two orders of magnitude and open up new possibilities in measurements using SMS.

Here we suggest a new method to improve signal rates significantly and suppress electronic scattering by orders of magnitude more than a HRM by using a very fast shutter combined with a microfocused synchrotron beam. By placing a shutter after a sample or material containing the nuclear resonant isotope, one can protect the detection system during the synchrotron excitation pulse by having a closed shutter. Then, having the shutter open in a time that is small compared with the nuclear level lifetime would allow one to detect the nuclear resonant emission without any non-resonant scattering. The difficulty in doing this is that a shutter would have to have a very fast transition time, *i.e.* the time from fully closed to fully open would have to be of the order of 10 ns. In addition, the shutter would have to sustain a repetition rate that matches the synchrotron pulse frequency. Depending on the time structure of the X-ray pulses delivered by a synchrotron source, this implies a repetition rate in the range 10^5^–10^7^ Hz. Also, the attenuation would have to be sufficient for the detector to withstand the prompt transmitted non-resonant radiation along with the delayed nuclear resonant radiation. In practice, this implies an attenuation of at least six orders of magnitude for present-day synchrotron beamlines. There are also benefits to increasing the attenuation significantly further. Specifically, complete attenuation of all the electronic scattering would allow, in addition to time-domain measurements, the production of a pure beam of Mössbauer photons that can be used for SMS in the energy domain or for other ultra-high-energy-resolution measurements with X-rays. Applications of SMS using X-ray free-electron lasers will produce a prompt X-ray flash owing to electronic charge scattering that is orders of magnitude larger than what is currently dealt with at third-generation synchrotron sources. This will overwhelm many of the current detection methods, but a high-speed shutter in combination with a microfocused beam has the potential to mitigate this problem.

## 2. Feasibility test

**Figure 1:**
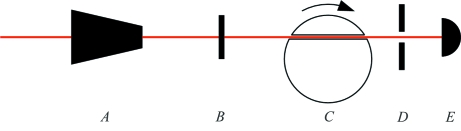
Measurement set-up. K–B mirror system (*A*), nuclear resonant foil (*B*), high-speed shutter (*C*), clean-up slits (*D*), APD timing detector (*E*). The X-ray beam was focused to the centre of the tunnel in the rotating shutter.

We performed a feasibility test at the BioCARS 14-ID beamline of the Advanced Photon Source. The storage ring was operated with a ‘hybrid fill pattern’, which has a single X-ray pulse followed 1.594 µs later by a 493 ns-long segmented pulse-train (http://www.aps.anl.gov/Facility/Storage_Ring_Parameters). This pattern repeats with a frequency of 271.554 kHz (period of 3.6825 µs). The single X-ray pulse originates from an electron bunch in the storage ring that produces 16 mA of stored current. The synchrotron radiation was filtered to a bandwidth of 1.9 eV (FWHM) at 14.4125 keV (corresponding to the first nuclear level in ^57^Fe) using a cryogenically cooled silicon double-crystal monochromator. The beam passed through a Kirkpatrick–Baez (K–B) mirror system to focus the beam and remove higher spectral harmonics. After the mirror system, but before the focal spot, the beam passed through a nuclear resonant material: 3 µm α-Fe or 12 µm stainless steel (composition 55% Fe, 25% Cr, 20% Ni), both enriched with 95% ^57^Fe. The K–B mirror system was operated so that the focal spot of 30 µm (vertical) by 100 µm (horizontal) was located approximately at the midpoint of a fast shutter system (Cammarata *et al.*, 2009 ▶). The rotation axis of the periodic shutter was orthogonal to the X-ray beam. This produced a vertical shutter speed transverse to the beam of 520 m s^−1^ and a duty cycle of 987.5 openings per second (once per rotation) of a tunnel in a spinning rotor, which closes/opens about the beam from top and bottom. Vertical and horizontal clean-up slits after the shutter were used to restrict the beam size to values smaller than the focal spot size. An APD with time-filtering electronics was used for time-differential measurements, producing time spectra with zero representing the arrival time of the incident X-ray pulse. Fig. 1 ▶ shows a schematic of the measurement set-up.

**Figure 2:**
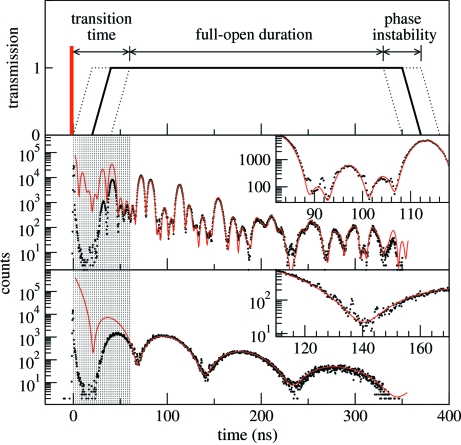
SMS measurements in the time domain using a fast shutter. The top panel shows the approximate transmission window of the shutter on a time scale; zero time corresponds to the X-ray excitation pulse. The middle panel shows the time spectrum of nuclear resonant decay in the forward direction of a 3 µm-thick α-Fe foil enriched with 95% ^57^Fe. The bottom panel shows the same for a 12 µm-thick stainless steel foil enriched with 95% ^57^Fe. Solid lines are simulations assuming the shutter opens immediately and help to demonstrate that the transmission window is fully open 60–330 ns after nuclear excitation. Insets are magnified regions that show contamination from spurious X-ray pulses.

Adjusting the phase of the rotating shutter such that its transmission window began after the arrival of the single X-ray pulse allowed a direct measure of the nuclear resonant signal from the α-Fe foil. With a 20 µm (vertical) slit after the shutter, we detected 54 counts s^−1^ in a time window of 60–330 ns after the excitation pulse. The expected energy-integrated transmission for this time window is 7.9Γ_0_ as obtained from the *CONUSS* software package (Sturhahn, 2000 ▶), where Γ_0_ = 4.67 neV is the width of the nuclear excited state. A time spectrum of the nuclear resonant emission in the forward direction for the α-Fe foil is shown in Fig. 2 ▶. Hyperfine fields at the nucleus produce nuclear level splittings that result in different resonant transition energies interfering and producing temporal beating that dominates the time spectrum. At our placement of the transmission window the shutter suppressed the excitation pulse by approximately 2 × 10^−9^ resulting in a counting rate of non-resonant scattering (at zero time) of 8.5 counts s^−1^. A very similar measurement of the stainless steel foil produced a time spectrum that is also shown in Fig. 2 ▶. The time spectrum also shows temporal beating, but this is due to multiple scattering that occurs in thick materials. We detected 11.5 counts s^−1^ in a time window of 60–330 ns after the excitation pulse, while the counting rate at zero time was 3 counts s^−1^. The expected energy-integrated transmission for this stainless steel foil is 1.7Γ_0_.

**Figure 3:**
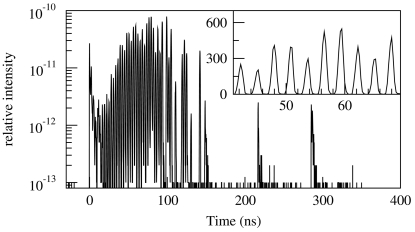
Measurement of contamination within the transmission window owing to spurious X-ray pulses from the storage ring. Intensities are relative to the X-ray excitation pulse. Spurious pulses are suppressed during the transition time (0–60 ns) of the shutter. The inset shows a magnified region of raw data in counts and clearly displays the 2.8 ns period of the storage ring’s RF. Data collection time was 15 h.

During initial testing we obtained a time spectrum without any nuclear resonant material to measure the background owing to spurious X-ray pulses. This was with a slightly larger slit opening (30 µm). This is shown in Fig. 3 ▶ and produced an integrated counting rate of 0.8 counts s^−1^ within a time window that starts 20 ns after the excitation pulse. These spurious pulses arrive at integer multiples of the storage ring’s RF period (2.8 ns) after the main X-ray pulse, and are due to electrons in the storage ring that occupy stable orbital positions other than those of the main electron bunches. This measurement of the spurious pulses cannot substitute for a proper measurement of the background but is representative of the nature and magnitude of the background lying within the transmission window. The actual background to the time spectra of Fig. 2 ▶ would be somewhat less owing to smaller slits and absorption in the foil; we estimate it to be approximately 0.2 counts s^−1^ in a time window of 60–330 ns after the excitation pulse.

For SMS measurements the shutter’s performance depends critically on its attenuation, overall transition time and full-open duration. The nuclear resonant measurements demonstrate excellent attenuation (10^−9^) of the electronic scattering owing to the excitation pulse, and a full-open duration of approximately 270 ns. The overall transition time can be estimated from the time spectra as that time after the excitation pulse when the nuclear resonant signal is no longer suppressed by the shutter. The overall transition time is approximately 60 ns and has two principal contributions: phase instability of the transmission window and the time for the shutter’s edge to traverse the microfocused beam. We measured the phase instability of the shutter’s transmission window using the 493 ns-long segmented pulse-train. We reduced the beam size to approximately 1 µm and collected time spectra of the pulse-train. By this we effectively made the one-shot transition time small (<1 ns) and were able to assess the long-term (20 min) phase instability from the closed-to-open time duration reflected in those time spectra. From this procedure we estimated the phase instability as reflected in the movement of the transmission window to be approximately ±20 ns. The measured phase instability has a fast component (jitter) that is reportedly 2 ns r.m.s. (Lindenau *et al.*, 2004 ▶) and a slow component that clearly dominates. In addition to the phase instability, the time to traverse a microfocused beam contributes to the overall transition time and is estimated to be approximately 20 ns for a 20 µm beam size.

## 3. Discussion

**Figure 4:**
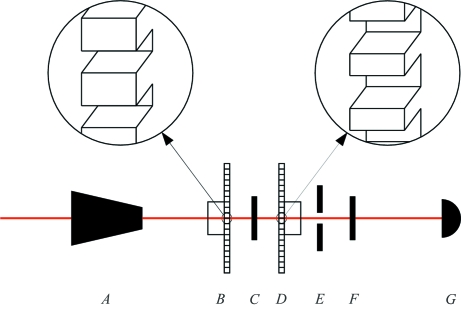
Proposed set-up for performing SMS using high-speed shutters. Axes of shutters are parallel to the X-ray beam. Focusing system (*A*), anti-shutter (*B*), sample or near-single-line resonant absorber (*C*), shutter (*D*), slits (*E*), sample or near-single-line resonant absorber (*F*), detector (*G*). For time-domain measurements a nuclear resonant sample is placed at position *C*, while nothing is placed at position *F*, and the detector needs a nanosecond time-resolution. For energy-domain measurements a near-single-line resonant material is mounted on a velocity transducer at position *C* (*F*), while the sample is placed at *F* (*C*), with the detector not requiring time resolution.

The measurements demonstrate the clear potential of using a fast shutter to perform SMS. There are two losses in the current set-up that could be removed in a future implementation: low duty cycle of the shutter and large focal spot. Operating at a higher duty cycle with an alternate shutter design would give a signal rate increase of as much as 275. This could be achieved by using a multi-slotted disc with its rotation axis parallel to the X-ray beam as shown in Fig. 4 ▶. Operating with a smaller focal spot would contribute by eliminating the loss at the slit (a factor of 1.5) and allow access to earlier time after the excitation pulse where more of the signal resides (a factor of 2). These factors alone would increase the measured signal rates for our two test materials by 825, which would lead to signal rates that are much higher than has ever been demonstrated for comparable resonant foils. This still assumes that one uses only 16 mA of the 100 mA of electron current in the storage ring. Further gains would be possible by using other storage-ring fill patterns and by improving high-speed shutter technology to achieve shorter transition times by reducing both phase instability and shutter traversal-time.

As shown in the insets of Fig. 2 ▶, spurious X-ray pulses contaminate the measurement and limit the usefulness of the technique for low-signal-rate applications. From the observed counting rates we estimate that the electron bunches responsible for the spurious X-ray pulses contain an average of approximately ten electrons. This translates to a bunch purity of 10^−11^ for the individual spurious pulses relative to the 16 mA excitation pulse. As this is already very good, it is unlikely that one will be able to suppress the background adequately for low-signal applications by improving the bunch purity within the storage ring.

This gives considerable impetus for employing a second shutter upstream (but still near a focal spot) of any nuclear resonant medium to suppress spurious X-ray pulses by being closed when the downstream shutter is open: an *anti-shutter*. Such an anti-shutter could also serve to alter the time period between excitation pulses and thus allow measurements to be performed using different timing modes that are implemented at various synchrotrons. The attenuation required for suppression of spurious pulses is quite moderate (15 attenuation lengths), while that for altering the excitation period would be substantially more (35 attenuation lengths). Note that a rotating shutter with greater attenuation will be more massive (thicker) and thus necessitate slower shutter speeds so as not to exceed maximum allowed tensile stresses.

Substantial suppression of both excitation and spurious pulses would allow one to produce a new source for Mössbauer studies. By removing the prompt electronic scattering from a thin nuclear resonant absorber one can produce a polarized X-ray beam with a Lorentzian spectral profile that approaches that of the nuclear level. In the case of ^57^Fe, a near-single-line resonant absorber, such as potassium ferrocyanide [K_4_^57^Fe(CN)_6_], placed on a velocity transducer between a shutter–anti-shutter pair would result in a new Mössbauer source that could be used to perform SMS in the energy domain. The velocity transducer would allow scanning the incident beam in energy through Doppler shifting in the same manner as in traditional Mössbauer spectroscopy.

The shutter will truncate the time response of the nuclear resonant emission, and this will modify the spectral composition of the X-ray beam after the shutter. This must be considered in order to produce a useful Mössbauer source. Assuming the shutter suppresses the electronic charge scattering completely, the spectral distribution *I*(ω) of the X-ray beam after the shutter owing to the transmission window (assuming a single Lorentzian-shaped resonance of width Γ in the thin resonant absorber limit) is given by
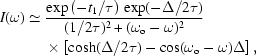
where *t*_1_ and Δ are the beginning and duration of the time window, respectively, while ω, ω_o_ and τ = 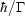 are the spectral frequency, resonant frequency and mean lifetime, respectively. In the limit of a thin resonant absorber, the spectral shape is affected by the width of the time window, but not the starting time. The starting time of the transmission window only affects the intensity. This would not be true for a thick resonant absorber owing to multiple scattering. Therefore, in order to produce a source with an acceptable spectral profile, it is important that both a thin resonant absorber be used and a time window of three to six mean lifetimes be available to avoid excessive spectral sidebands owing to an artificially truncated time response.

Fig. 4 ▶ shows a two-shutter set-up suitable for SMS measurements in either the time domain or energy domain. For energy-domain measurements one could enhance the detection rate by using a much more efficient X-ray detector in place of a fast-timing detector, which is typically less efficient. This modified set-up would allow the production of a pure Mössbauer beam with a spectral width similar to that of a traditional radioactive source, but without the unwanted spectral components that emanate from radioactive materials originating from electronic and nuclear fluorescences. Also, it would allow polarization control and produce many orders of magnitude more spectral brightness owing to the much greater collimation and much smaller beam size. A near-single-line synchrotron Mössbauer source has been produced previously using pure nuclear Bragg diffraction from a ^57^Fe-enriched single crystal of iron borate (^57^FeBO_3_) (Smirnov *et al.*, 1997 ▶) and continues to be improved upon with good results (Mitsui *et al.*, 2009 ▶), but high-speed shuttering offers the potential for a larger source strength and the possibility for use with other resonant isotopes. This would allow nuclear Bragg/Laue diffraction from crystalline or polycrystalline samples to be performed with greater practicality. Also, measurements of microscopic samples would benefit enormously over traditional Mössbauer spectroscopy owing to the enhanced intensity associated with a focused beam.

The primary restriction of the proposed scheme is the need for a microfocused beam in at least one dimension, along the direction of shutter motion. The position of this one-dimensional focus dictates the location of the shutter–anti-shutter pair. In a time-domain set-up where one measures time spectra as in Fig. 2 ▶, the sample could be placed at the focal spot (position *C* in Fig. 4 ▶) with the shutter and anti-shutter immediately downstream and upstream, respectively, of the sample environment. In the energy-domain set-up there are multiple possibilities. One option would be to place a near-single-line resonant absorber on a velocity transducer at the focal spot (position *C*), while the sample environment would be located immediately after the downstream shutter (position *F*), where the beam size will be larger (in one dimension) but with otherwise little restriction on the sample environment. Alternatively, the sample could be placed between the shutter–anti-shutter pair with a near-single-line resonant absorber placed after the downstream shutter on a velocity transducer. This has the advantage of a small beam at the sample and the possibility to perform both time-domain and energy-domain measurements coincidently, but has the drawback of a lower ‘resonant absorption effect’ (*i.e.* signal-to-background ratio) in the energy spectra. Note that the location of the focus associated with the orthogonal beam dimension is not significantly restricted and may be dedicated to the sample environment in either the time-domain set-up or the energy-domain set-up.

Although two-dimensional microfocusing is unnecessary in principle, it presents significant advantages in practice. In particular, a small beam (in both transverse dimensions) eases the restrictions on high-speed shutter design. For third-generation synchrotron sources, a high-speed shutter composed of a metal disc with a rotation axis parallel to the X-ray beam will need openings with a periodic spacing of 100 µm to 1 mm. The actual spacing will depend on the precise time structure of the synchrotron pulses and operating parameters for the rotating disc. For high-repetition-rate operation, the periodic shutter spacing can become sufficiently small (*e.g.* 100 µm) that one might need micromachining methods to fabricate the shutter openings. In this case, two-dimensional focusing would allow acceptable aspect ratios for the micromachined features.

Currently, fast shutters used for X-ray beams that use rotating metal discs (0.5 mm-thick titanium alloy) with periodically spaced openings have demonstrated maximum tangential speeds in excess of 1000 m s^−1^ (Lindenau *et al.*, 2008 ▶). The time for an ideal shutter edge of such a device to traverse a microfocused beam of 10 µm would result in a traversal time of 10 ns. Smaller focal spots would produce even shorter traversal times. Also, one has to combine this traversal time with the phase-instability time to obtain the actual transition time from fully closed to fully open. Various X-ray switching techniques for other applications have demonstrated very fast transition times from closed to open, but are inadequate for SMS owing to low transmission when open and inadequate suppression when closed (Grigoriev *et al.*, 2006 ▶; Tanaka *et al.*, 2002 ▶). Improving high-speed shutter technology towards greater phase stability of the transmission window (for multi-slotted discs) will produce even higher signal rates and increase its suitability for short-lived resonant isotopes. A high-speed shutter is best suited for low-repetition-rate sources involving very high instantaneous pulse intensities. Consequently, this method can be employed for applications of SMS using an X-ray free-electron laser to suppress the enormous quantity of electronic scattering that would otherwise overwhelm conventional methods.
